# 

**Published:** 1877-09

**Authors:** 


### Contents of September Number.

### ORIGINAL.

ART. I.—Certain Points in Diphtheria, by F. C. Curtiss, M. D., Albany, 41
ART. II.—Clinical Lecture on Angular Curvature; Its Pathology and Treatment, by Prof. J. F. Miner, M. D. Reported by Chas A. Wall, B. S., .... 49
ART. III.—Medico-Legal Facts and Testimony in the Trial of Henry C. Hendryx, for the murder of his wife, by J. C. Young, M. D., of Cuba, Allegany Co., .... 57

### CORRESPONDENCE.

Druggists as Prescribers, .... 59

### MISCELLANEOUS.

Cost of Maintenance of the Insane, by H. B. Wilbur, M. D., .... 62
The Germ Theory Controversy, .... 67
Recent Progress in Obstetrics and Gynæcology, by S. Howe, M. D.
Is the Fœtus in Utero affected by Medicine given the Mother, .... 68
Face Presentations, .... 70
Influence of Posture on Women, .... 71
The Passage of Salicylic Acid and Iodide of Potash from the Mother into the Liquor Amnii, .... 71
New Forceps, .... 71
Instrumental delivery without the knowledge of the patient, .... 72
The Production of Albuminuria, .... 72

### EDITORIAL.

Advertising by Physicians, .... 73
Transactions of the Alumni Association, .... 73
Meeting of Association for cure of Inebriates, .... 74
The Popular Science Monthly, .... 74
BOOKS REVIEWED:
Civil Malpractice, by Milo A. McClelland, M. D., .... 75
Ziemissen's Cyclopædia, Vol. XII. Diseases of the Brain and its Membranes, .... 77
Books and Pamphlets Received, .... 80

